# 

**Published:** 1895-03

**Authors:** 


### Society Meetings.

The American Academy of Medicine will hold its twentieth annual meeting in one of the buildings of the Johns Hopkins University on Saturday, May 4th, and on Monday, May 6, 1895. The headquarters of the Fellows of the academy and the meetings of the council will be at the Stafford.

The meeting will open at 10 o'clock on Saturday morning with an executive session of the Fellows of the academy exclusively ; the reading of the papers will begin at about 11. The morning session will close at 1 o'clock and the session of Saturday afternoon will extend from 3 to 6. The " Reunion session " will be held on Saturday evening. By a standing rule the price of the tickets for the supper is fixed at $2. Attendance at the reunion session is not confined to the Fellows exclusively, hence any member may bring friends with him by arranging for their tickets with the committee. For the past two years ladies have been present at this session and have added to the enjoyment. The session of Monday will begin with a short executive meeting, after which the reading of papers will be resumed ; after a recess at 1, the afternoon session will begin at 3 and continue until adjournment.

Members of the profession and others who may be interested in the topics treated by the papers, are cordially invited to attend the open sessions of the academy.

An interesting program has been prepared, among which we notice the following titles by physicians at Buffalo: The proper teaching of Physiology in the Public Schools as a means of preventing Intemperance and Venereal Disease, by De Lancey Rochester, M. D.; What shall we do with our Alcoholic Inebriates? by J. W. Grosvenor, M. D.

---

### Board of Health Note.

PROFESSOR B. MEAD BOLTON, associate professor in bacteriology at Johns Hopkins University, was appointed by the Buffalo Civil Service Commission to conduct the examination of the applicants for bacteriologist and assistant bacteriologist to the health department of Buffalo. This examination was held on Friday and Saturday, February 15 and 16, 1895. Seven applicants contested for the honor. Of these, two, Drs. William G. Bissell and Thomas B. Carpenter, who have been performing the duties of the offices pending the result of this examination, obtained the highest markings and will undoubtedly be appointed permanently to the places they have been holding.

### Journalistic Note.

THE *Odontographic Journal*, a dental quarterly edited by J. Edward Line, of Rochester, and published by the Rochester Dental Manufacturing Company, has appeared in a handsome new cover. It is well edited and printed and presents unmistakable evidences of prosperity.

Scientific Bibliography.—Mm. J. B. Bailliere & Son have published a new General Catalogue of Scientific Books, (medicine, natural history, agriculture, physics, chemistry, arts and manufactures,) forming a volume of 112 pages, octavo, two columned.

This bibliography contains the detailed announcement of more than 5,000 volumes, preceded by a table of contents arranged in alphabetical order and will be sent gratis to each of the readers of the Journal who will send their request to Mm. J. B. Bailliere & Son, 19 rue Hautefeuille, à Paris.

---

Messrs. Parke, Davis & Co., of Detroit, were favored with an unsolicited visit by a representative of the *Detroit Journal* to their new bacteriological laboratory. A full account of this call is published in the *Journal* February 9, 1895, giving in considerable detail the methods of the manufacture of antitoxin pursued in that laboratory. This firm has recently published and sent out to the profession a beautiful brochure on the pharmacology of kola. It contains a few well-executed colored plates, showing the growth of the kola nut from the blossom to maturity. This pamphlet will be furnished to physicians on application.

---

THE *Monatsschrift für Geburtshülfe und Gynäkologie* is the name of a new medical magazine published in Berlin and edited by Professor Dr. A. Martin in Berlin, and Professor Dr. M. Sänger in Leipzig. Volume I., Number 1, January, 1895, is a handsomely printed 96 pp. magazine, which has been sent to us through the courtesy of B. Westermann & Co., 312 Broadway, N. Y. The editors are both Honorary Fellows of the American Association of Obstetricians and Gynecologists.

---

THE Antikamnia Chemical Co., of St. Louis, has issued a handsomely printed and bound monograph entitled Surgery 200 Years Ago. It is illustrated from original copper plates, showing crude methods of amputation, blood letting, reduction of dislocations, performance of lithotomy, trephining, transfusion and other surgical measures. This interesting book will be furnished only by the Antikamnia Chemical Co., and will be supplied to physicians upon application.

---

THE proprietors of Tongaline and Ponca Compound have just issued a neat and convenient Physician's Pocket Diary and Daily Memorandum Book, which contains much useful and valuable information for the general practitioner. It was the intention to have one in the hands of every physician in the United States by January 1st, but if through an error in addressing or negligence on the part of the post-office officials, any physician should not have received a copy, it will be mailed on application to the Mellier Drug Company, 2112 Lucas Place, St. Louis, Mo.

---

THE McArthur Hypophosphite Company, of Boston, has issued an artistic calendar for the current year that combines excellence of taste with practical usefulness.

